# Supplementary figures and images for: Pore-forming alpha-hemolysin efficiently improves the immunogenicity and protective efficacy of protein antigens
Source: PLoS Pathog. 2021 Jul 21;17(7):e1009752. doi: 10.1371/journal.ppat.1009752 (PMC8294524; doi:10.1371/journal.ppat.1009752)

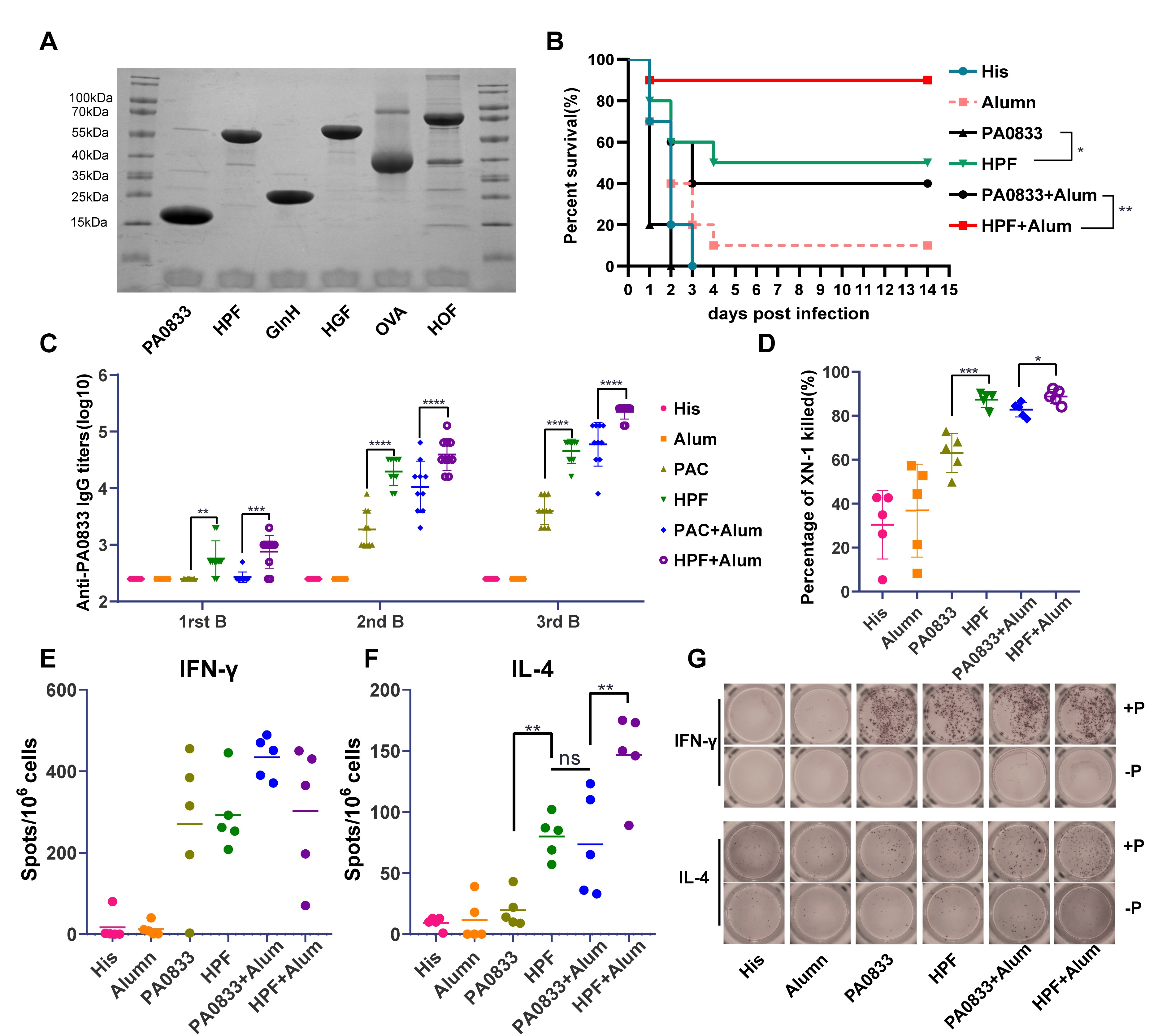

Supplement: S1 Fig — (A) SDS-PAGE analysis of PA0833, HPF, GlnH, HGF, OVA and HOF. (B) One week after the last immunization, mice were challenged with 3 × 10^7^ CFUs of P. aeruginosa strain XN-1 and monitored continuously for 14 days (n = 10). Log-rank (Mantel-Cox) test, *P<0.05, **P<0.01. (C) Titers of PA0833-specific IgGs from immunized C57BL/6 mice were determined by indirect ELISA after the first, second, and third immunization (n = 10). Two-way ANOVA, Tukey’s multiple comparison test, **P<0.01, ***P<0.001, ****P<0.0001. (D) The P. aeruginosa strain XN-1 was tested for survival in serum from different immunization groups. Unpaired student’s t test, *P<0.05, ***P<0.001. ELISPOT analysis of IFN-γ (E) and IL-4 (F) secretion of splenocytes from different immunization groups. One-way ANOVA, Tukey’s multiple comparison test, **P<0.01. (G) Representative images from ELISPOT analyzes. (TIF) [file ppat.1009752.s001.tif]

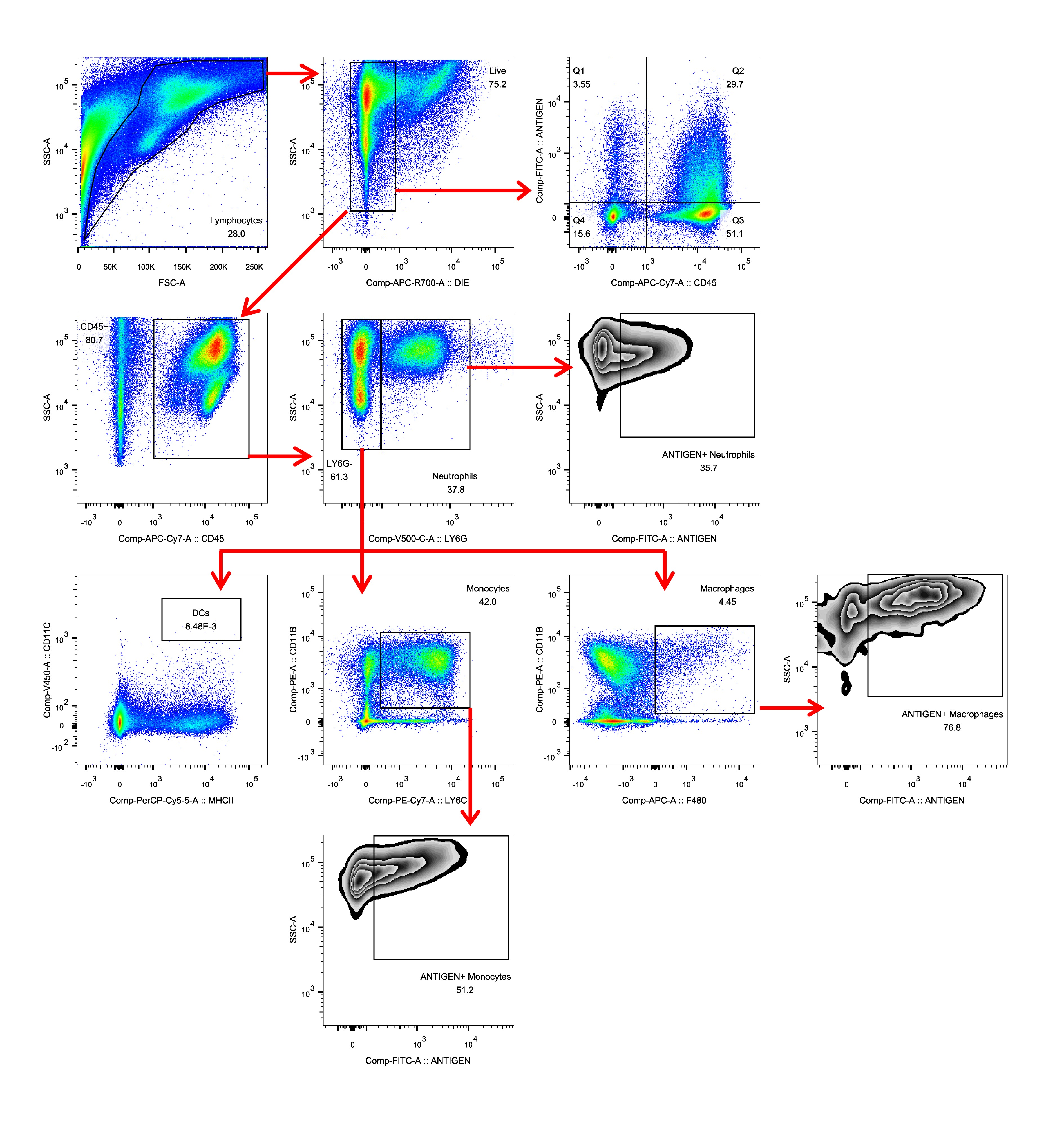

Supplement: S2 Fig — (TIF) [file ppat.1009752.s002.tif]

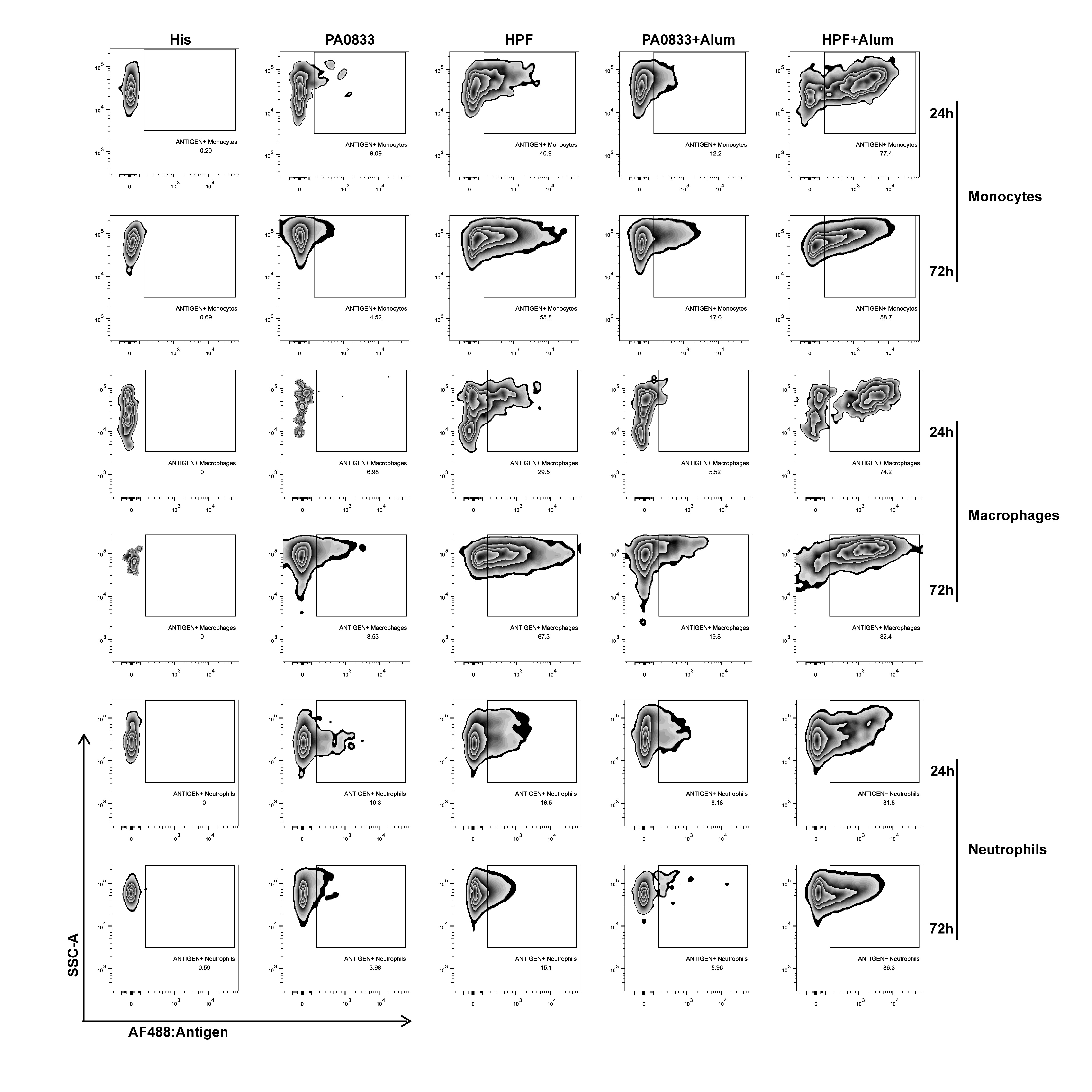

Supplement: S3 Fig — Zebra plot of antigen-positive monocytes, macrophages, and neutrophils 24 h and 72 h after injection. Alexa Fluor 488-labeled antigen-positive cells were gated against the His buffer control group. (TIF) [file ppat.1009752.s003.tif]

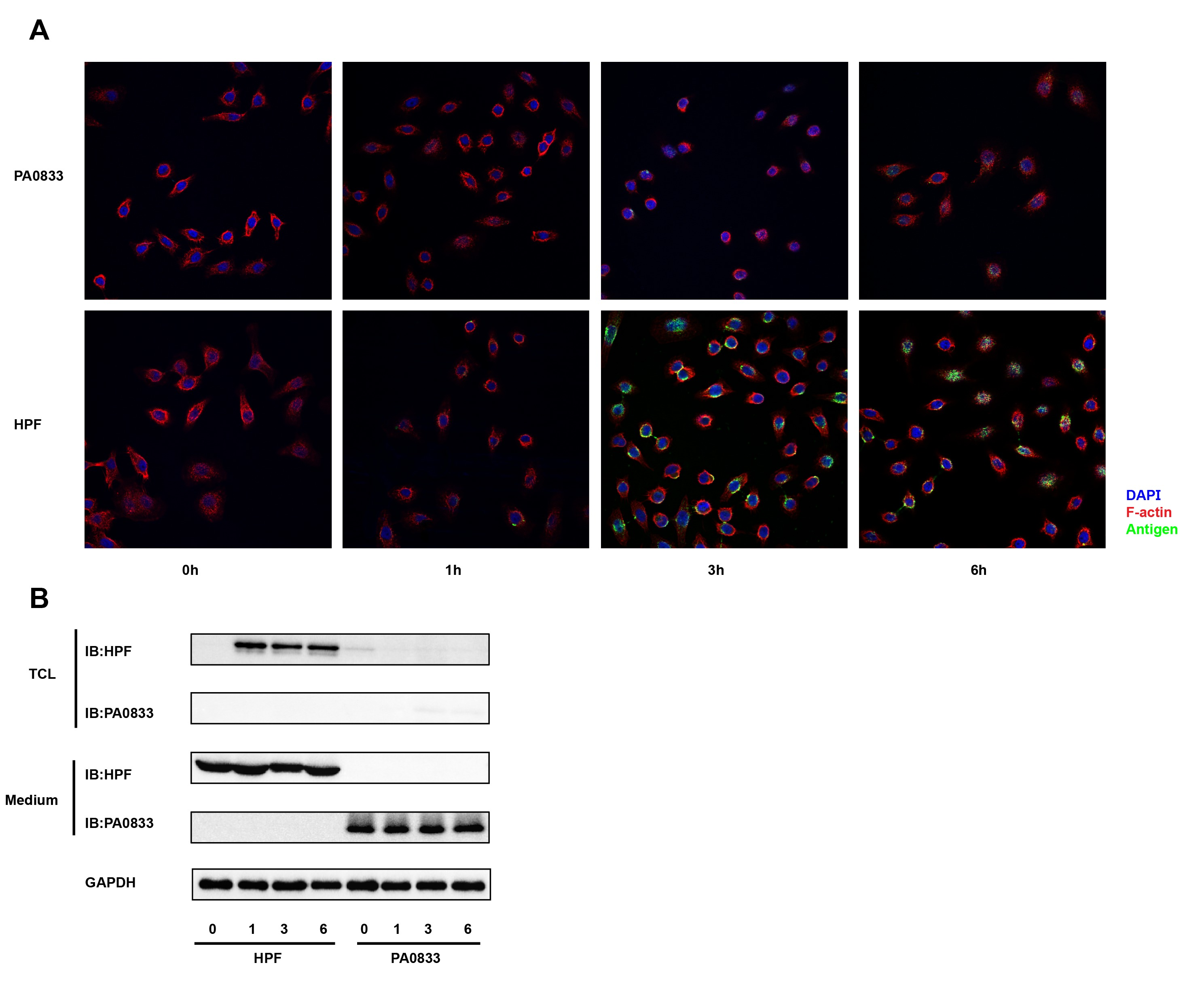

Supplement: S4 Fig — (A) Alexa Fluor 488-labeled PA0833 and HPF uptake by A549 cells was captured by confocal microscopy. (B) After the indicated incubation, PA0833 and HPF uptake by THP-1 cells was determined by immunoblotting. (TIF) [file ppat.1009752.s004.tif]

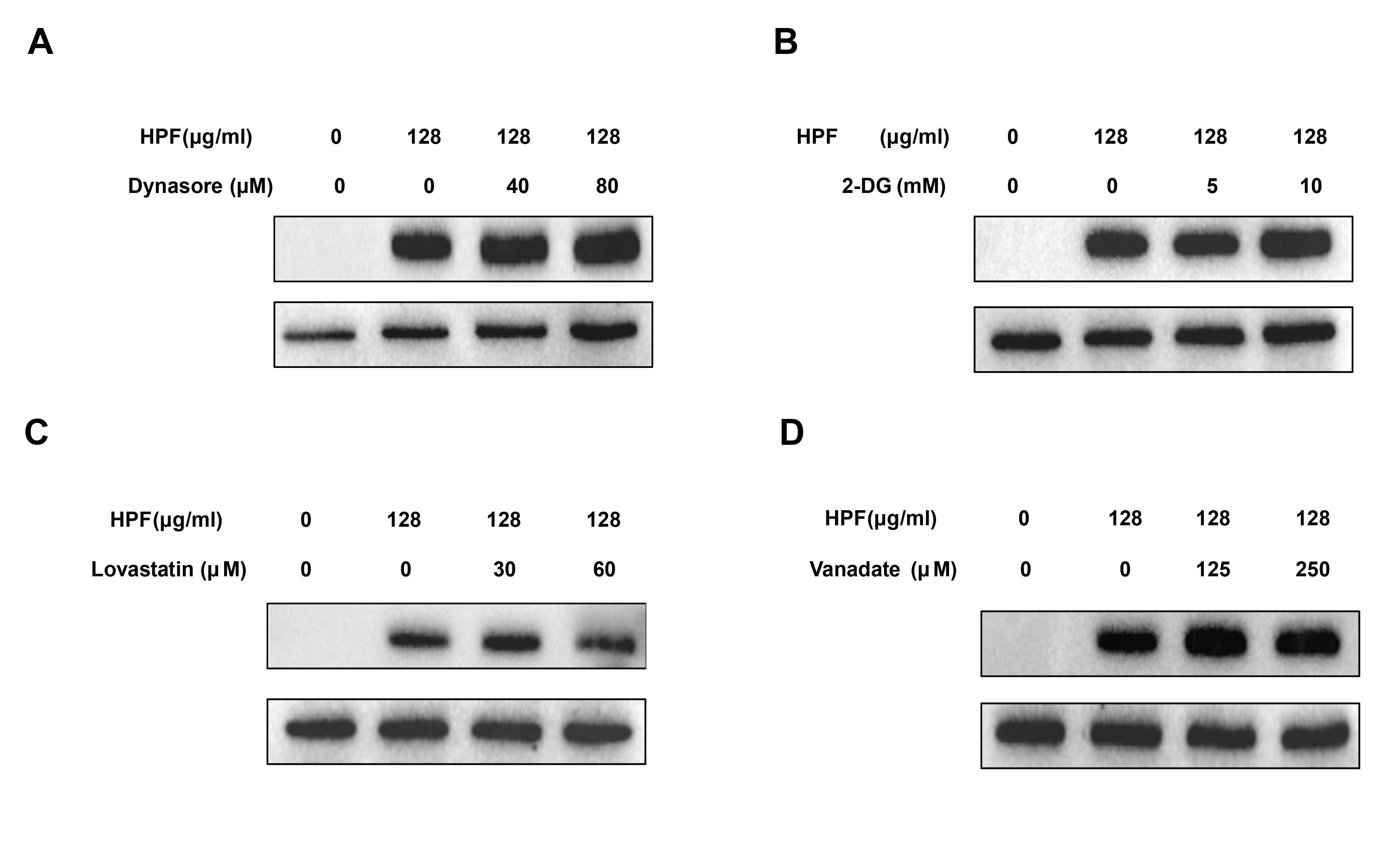

Supplement: S5 Fig — After pretreatment with the indicated concentrations of dynasore (A), 2-deoxy-D-glucose (2-DG)(B), lovastatin (C), and vanadate (D), complete HPF uptake by RAW264.7 cells was determined by immunoblotting. (TIF) [file ppat.1009752.s005.tif]

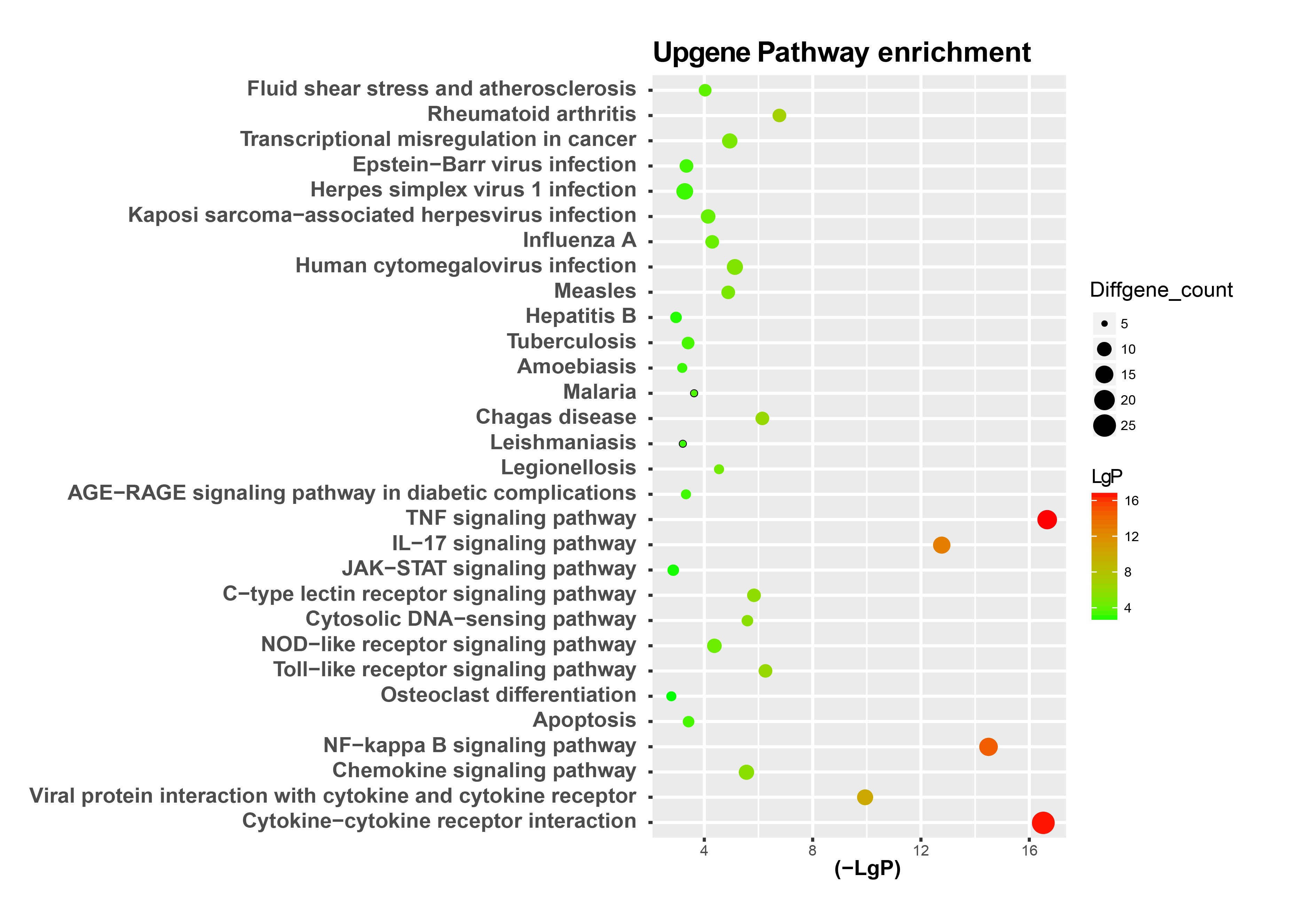

Supplement: S6 Fig — (TIF) [file ppat.1009752.s006.tif]

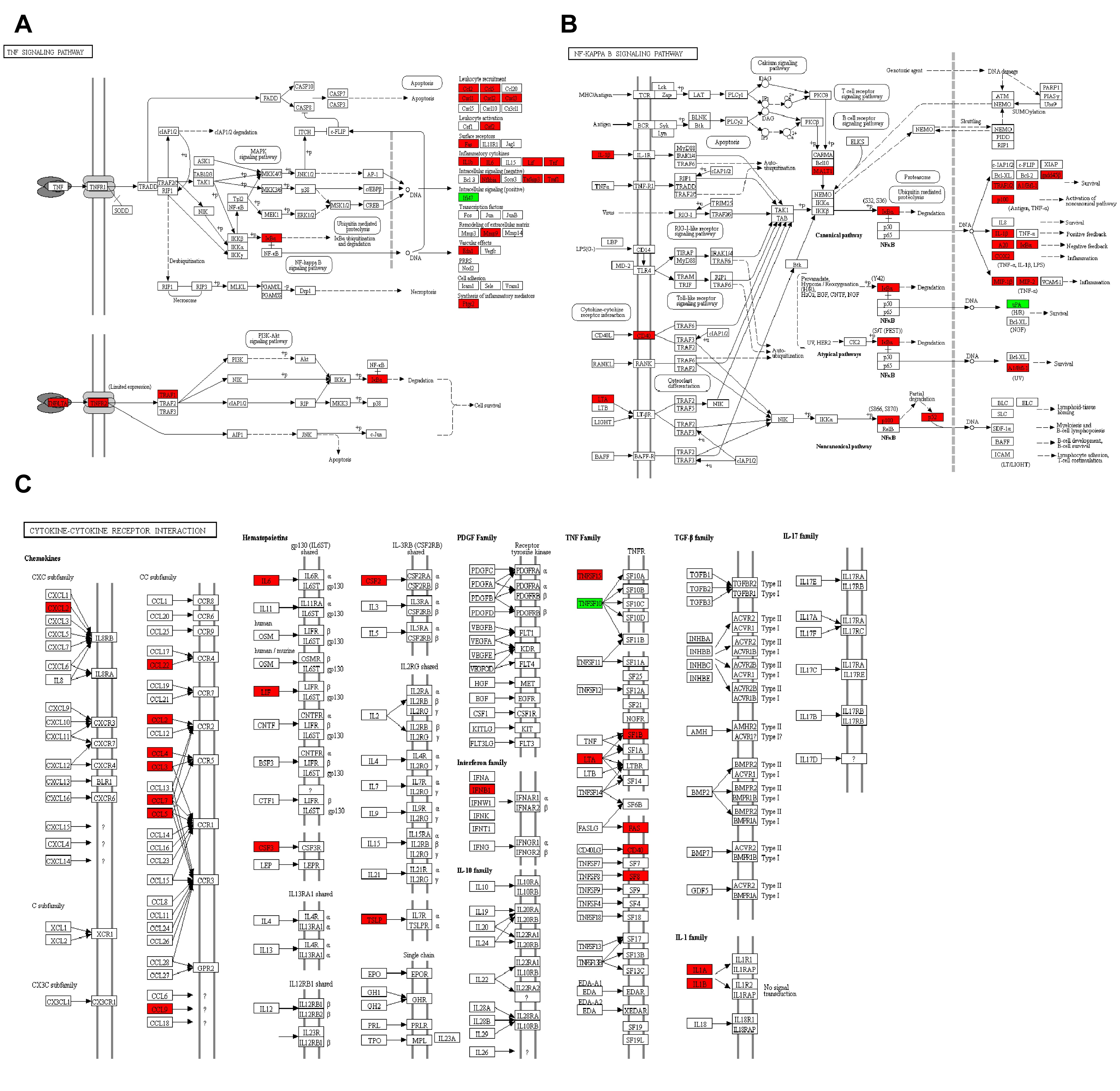

Supplement: S7 Fig — Schematic representation of differentially expressed genes enriched in TNF (A), NF-κB (B) and cytokine-cytokine receptors interaction (C) signaling pathway. Red, white and green color represents up-regulated genes, no difference genes and down-regulated genes, respectively. (TIF) [file ppat.1009752.s007.tif]
