# Supplementary material for: Pore-forming alpha-hemolysin efficiently improves the immunogenicity and protective efficacy of protein antigens
Source: PLoS Pathog. 2021 Jul 21;17(7):e1009752. doi: 10.1371/journal.ppat.1009752 (PMC8294524; doi:10.1371/journal.ppat.1009752)
Supplement: Attachment — Submitted filename: Response_Letter2 .docx [file ppat.1009752.s010.docx]

Dear Editor:

Thank you for giving us an opportunity to revise our manuscript titled “Pore-forming alpha-hemolysin efficiently improves the immunogenicity and protective efficacy of protein antigens” (PPATHOGENS-D-21-00434). The comments from the reviewers are important for us to improve the overall quality of our manuscript, and provide important guidance for our future research. We have addressed all reviewers’ comments, the point-to-point responses to the comments are listed as follows. Meanwhile, we will submit the revised text with tracked changes, corrected figures with the Preflight Analysis and Conversion Engine (PACE) digital diagnostic tool, and supplement files.

**Reviewer #1**

**PartⅠ- Summary**

In this manuscript “Pore-forming alpha-hemolysin efficiently improved the immunogenicity and protective efficacy of protein antigens”, Zou et al describe the use of a mutated form of the S. aureus protein Hla as a protein carrier for fusion to antigens used for immunization. The data suggests that the fusion proteins, when combined with alum, can offer substantial protection (100% survival and over 3 log reduction in bacterial burden) from infection with P. aeruginosa. The authors go on to show that the Hla-fusion proteins can induce robust antigen-specific IgG responses, in addition to cytokine responses. They show that antigen uptake occurs by monocytes, macrophages and neutrophils all uptaking the antigen. Overall, this is a concise and well-written manuscript with robust data.

**Response:** Thank you for your encouraging remarks and valuable comments. We have corrected our text and performed assays according to your comments. Our responses to your comments are detailed at Major Issues and Minor Issues parts.

**Part II – Major Issues: Key Experiments Required for Acceptance**

The authors did not compare their HPF to one of the well-established protein carriers like diphtheria toxin or tetanus toxoid to test if Hla is indeed a superior protein carrier than molecules with known safety profiles. Without these data, it is hard to gauge the potential impact of the presented findings.

**Response:** Thank you for your constructive suggestion. Due to the poor immunogenicity of some polysaccharide antigens, diphtheria toxin and tetanus toxoid are wildly used as carrier proteins to improve the immunogenicity of polysaccharides. The toxin-polysaccharide conjugates are usually obtained by chemical means; however, these methods cannot be easily applied for protein-protein conjugates. Thus, there is little study on the impact of these toxins in improving the immunogenicity of protein antigens. In this study, the Hla^H35A^-PA0833 fusion protein was obtained by linking the DNA sequences encoding the two proteins together, which was then expressed as a fusion protein. In response to your suggestion, we have tried to get the fusion protein of diphtheria toxin and tetanus toxoid linked with PA0833. However, we have not got the soluble proteins yet, probably due to the large size of the proteins.

The lack of DC recruitment to the injection site is puzzling and may need to be further investigated.

**Response:** Thank you for your concerns. In fact, the recuitement of immune cells by alum and MF59 in injected muscle has been previously examined in immunized BALB/c mice (Samuele et al., 2010 DOI: 10.1016/j.vaccine.2010.12.090). Samuele Calabro et al showed that alum and MF59 mainly induce recruitment of neutrophils and monocytes rather than DCs, consistent with the results in Rhesus macaques model (Liang F et al., 2016 DOI: 10.1126/scitranslmed.aal2094), these results were in consistent with our results.

**Part III – Minor Issues: Editorial and Data Presentation Modifications**
Could the authors use and ADAM10 inhibitor and show that the response to the fusion protein is diminished, therefore directly linking the increased response of using Hla to the binding of ADAM10?

**Response:** ToADAM10 specific inhibitor, GI254023X, was used to determine the function of ADAM10 on HPF uptake by monocytes. The result was presented at Figure 4F and the corresponding text was corrected.

**Reviewer #2**

**Part Ⅰ- Summary**

This study by Zou and colleagues tests a novel hemolysin and pseudomonas antigen fusion protein as a novel vaccine platform. The strength of the study is mainly in the innovation of this construct. The data in mice show increased antibody responses compared to immunization with the pseudomonas antigen alone and also impressive enhancement of protective immunity, but only in the presence of alum. The protective immunity is not improved without alum as shown in Figures 1C and 1D. In addition, to achieve this effect three immunization are necessary. The immunity is not tested against other pseudomonas antigen, only one mouse strain and one sex are tested. This limits the enthusiasm for the potential applicability of this platform. The cellular response studies in muscle and lymph nodes seem mostly dependent on the alum effect. Also, the conclusion that there is an increased Th2 response is only based on slightly increased IgG1 titers.The relevance of the in vitro studies is limited by the use of tumor cell lines.

**Response:** Thank you very much for your constructive suggestions.

First, the enhancement of protective immunity without alumn was confirmed upon re-analysis with Gehan-Breslow-Wilcoxon test. Please check at Figure 1C. Meanwhile, when this study was repeated in a lethal pneumonia model on male C57BL/6 mouse, significant difference was also observed in the absence of alum in the two groups. Thus, the protective effect of PA0833 is improved by Hla^H35A^.

Second, we are sorry for not comparing with other pseudomonas antigen. In this study, we are focused on confirming the effect of Hla^H35A^ on the improvement of immunogenicity of protein antigens, since PA0833 is an antigen from pseudomonas aeroginosa identified by us previously, we used this antigen first. After the effect of Hla^H35A^ was confirmed, we have tested the efficacy on two other protein antigens, including GlnH from *Klebsiella Pneumoniae* and classical antigen Ovalbumin (OVA).

Although they are not pseudomonas antigens, these results indicate Hla^H35A^ is efficient to improve the immunogenicity of protein antigens.

Third, we have repeated the experiment in Fig. 1 in a lethal pneumonia model on male C57BL/6 mouse. Third, we have repeated the experiment in Fig. 1 in a lethal pneumonia model on male C57BL/6 mouse. The results, which were consistent with previous findings, were prepared in Figure S1, and the text was also revised.

**Part II – Major Issues: Key Experiments Required for Acceptance**

Confirmation of the enhancement of protective and Th2 immunity in a different haplotype.

**Response:** Thank you for your comments, it is critical to test our strategy on other mouse strain. So, we have repeated the experiment in Fig. 1 in a lethal pneumonia model on male C57BL/6 mouse. The results was added into Figure S1B, C and D. IFN-γ and IL-4 ELISpot analysis (Figure S1E, F and G) of splenocytes stimulated with PA0833 was performed as previously described (Liang F et al., 2016 DOI: 10.1126/scitranslmed.aal2094), and Th2 response was confirmed by enhancement of secretion of IL-4.

Clearer delineation of the alum dependent responses.

**Response:** To clarify alum dependent responses, we added alum as a control group in experiment and added this result in Figure S1. As you can see, alumn alone exerted a slight increase of protective immunity compared to histine buffer, but the efficacy was limited.

**Reviewer #3**

**Part Ⅰ- Summary**

The authors described a fused protein as potential vaccine candidate. Several fused proteins including chimeric and trimeric vaccines were previously described. The authors did not show the advantage of their fused protein compared to other vaccine candidates against pseudomonas.

**Response:** Thank you for your concerns regarding our research. In this study, we try to focus on a workable solution for improve the immunogenicity of protein antigens. PA0833, which was previously identified as an antigen candidate from *P. aeruginosa*, was selected as a target to verify the adjuvant efficacy of Hla^H35A^. After this effect was confirmed, we then tested two protein antigens from other species (Figure 7) instead of other pseudomonas antigens to verify the universal efficacy of Hla^H35A^.

**Part II – Major Issues: Key Experiments Required for Acceptance**

Complement mediated killing has a major role in clearance of Gram negative bacteria including pseudomonas species. The authors did not show how complement functional activity affected before and after immunisation.

**Response:** We fully agree that it is necessary to detect the complement mediated killing. Hence, we performed the serum bactericidal assay, and result was presented in Figure S1D.

-The authors cloned 2 coding sequences in one expression vector. Would you please describe this in details. Did you express the fused protein under control of one promotor or your vector has two different promotors.

**Response:** In this study, the sequences encoding two proteins were obtained from National Center for Biotechnology Information (NCBI) and integrated into one sequence by a GGGGS linker. Then the whole sequence was codon optimized for *E. coli*. The optimized sequence was synthesized and cloned into the pGEX-6p-1vector. Thus the fusion protein was expressed under control of one promoter.

-The discussion section is poorly written and the authors did not high light other vaccine candidates against pseudomonas.

**Response:** We have revised the discussion section. In this study, we are focused on confirming the effect of HlaH35A on the improvement of immunogenicity of protein antigens, since PA0833 is an antigen from pseudomonas aeroginosa identified by us previously, we used this antigen first. After the effect of HlaH35A was confirmed, we have tested the efficacy on two other protein antigens, including GlnH from Klebsiella Pneumoniae and classical antigen Ovalbumin (OVA). Thus, the purpose of this study is not to develop a vaccine against pseudomonas aeroginosa, so the other vaccine candidates against pseudomonas were not discussed. However, if you think it is necessary, we would add this later.

**Part III – Minor Issues: Editorial and Data Presentation Modifications**

-The resolution of the figures is not good

**Response:** Sorry for the low resolution of these figures. In the revised version of this manuscript, all figures have been revised and corrected with the Preflight Analysis and Conversion Engine (PACE) digital diagnostic tool according to editors’ instructions.

- English language editing is also required in some section

**Response:** We feel sorry for our poor English language editing, and the revised version has been submitted for language edition by elsevier language editing.
